# Transfer of arthroscopic skills from computer simulation training to the operating theatre: a review of evidence from two randomised controlled studies

**DOI:** 10.1051/sicotj/2015039

**Published:** 2016-02-02

**Authors:** Tarek Boutefnouchet, Thomas Laios

**Affiliations:** 1 University Hospital Coventry and Warwickshire Clifford Bridge Road Walsgrave, Coventry CV2 2DX UK; 2 Warwick Medical School, The University of Warwick Coventry CV4 7AL UK; 3 Department of Trauma and Orthopaedic Surgery, Heart of England NHS Foundation Trust, Heartlands Hospital Bordesley Green East Birmingham B9 5SS UK

**Keywords:** Surgical training, Computer simulation, Haptic technology, Arthroscopy, Skills, Assessment

## Abstract

*Introduction*: There is paucity in the research on transfer validity of arthroscopic simulator training. The aim of this article is to determine whether skills derived from arthroscopic simulation are transferrable to the operating theatre and retained over time.

*Methods*: A systematic review with rigorous criteria to identify the highest level of evidence available was carried out. The studies were critically appraised with narrative data synthesis.

*Results*: Twenty-one studies on arthroscopic simulation were identified. Only two studies were randomised controlled trials. The first article demonstrated improved performance of basic knee arthroscopic tasks following a fixed period of training. The second article showed improved performance of arthroscopic tasks and no deterioration in the levels of skills following a period of six months. In addition, the two studies succeeded in demonstrating the importance of 3D motion analysis using computer simulators in the assessment of technical skills. Components of evaluation such as time to task completion, distance travelled by instruments and incidence of instruments collisions were associated with the highest validity and reliability of assessment. This systematic review highlighted the limitations of these two randomised studies.

*Discussion*: Evidence from the two trials suggests that knee arthroscopy simulator training can result in improved performance. This review helped highlight the contribution of the two studies in terms of internal validity and consistency of using arthroscopic skills training. Further level I studies are however required to demonstrate the evidence for transfer and predictive validity of computer simulation as a training instrument.

## Introduction

The aviation industry has often been proposed as a model for surgical training [1]. Both disciplines require a coordinated team effort often under pressure [2]. The demands of such tasks require pilots to spend time on flight simulators prior to real task exposure [3]. Novice pilots trained on flight simulator demonstrate better psychomotor skills compared to control groups [4]. Hays and colleagues showed better retention of skills following flight simulator training when compared to conventional in-flight training [5]. Equally, simulation in clinical training illustrates the link between doing and thinking and is compatible with the model derived from Kolb’s four stage experiential learning cycle [6]. Utilising such approaches allows learners to develop optimal clinical performance while providing a safe environment. In contrast, simulation has its limitations; arguably gaining popularity in recent times due to the impact of service provision and shortened training time [7]. Consequently, recent years have seen an expansion in the literature related to simulation training across various clinical specialities [8].

The traditional model of one-to-one apprenticeship has trained successive generations of surgeons across the world and still prevails. In more recent times however the surgical community at large started to recognise the limitations of this traditional system, especially with the change from time-based to competency-based training and the pressures from health service providers [9]. In addition, significant restrictions are placed on surgeons in training by the European Working Time Directives [10], a position reflected in the rest of the developed world [11]. Consequently, several institutions introduced cadaveric labs and simulators to palliate the current situation facing surgical training and implement lessons learned from the aviation industry. This method also came with the promise of added objectivity in assessing technical skills [12]. This was met with a growing plethora of research investigating the feasibility, reliability and validity of simulation-based surgical skills training [13, 14]. Equally, arthroscopic surgery demands a complex set of psychomotor skills with a particular focus on visual and spatial awareness [15]. Technical advent and the introduction of haptic technology brought a new sphere of possibilities in this field [16].

With an estimated incidence of 10 knee arthroscopic procedures per 10,000 population performed in the National Health Service (NHS) in England annually, there is clearly a need to consider the evidence for improved teaching modality for this specific skill set [17]. Two recent systematic reviews demonstrated a high level of internal consistency and validity using simulators for arthroscopy skills training. The same authors concluded that there is a need for further studies to demonstrate transfer and retention of skills following simulator training [18, 19]. Despite the growing body of evidence the main plethora of research, which has focused on transfer of skills from the simulator to the operating room, remain in the field of laparoscopic surgery [20, 21]. Equally, there is paucity in the research looking at the so-called “transfer validity” in arthroscopic simulator training [8, 18, 19]. Lack of transfer validity can potentially make simulation training dissociated from the reality of clinical practice. Therefore, the aim of this review was to identify and evaluate the best evidence available for improving arthroscopic surgical skills using simulation training among orthopaedic surgical trainees and whether these skills are transferrable to the operating theatre and retained over time. It is hypothesised here that surgeons trained on computer simulator for knee arthroscopy demonstrate better transfer and retention of psychomotor skills when evaluated with standard competence-based assessment and objective measures of psychomotor skills.

## Material and methods

A systematic review of the literature was performed according to the methods described in the Preferred Reporting Items for Systematic Reviews and Meta-Analyses (PRISMA) statement [22]. Literature searches were performed using terms related to computer simulation, virtual reality, orthopaedic surgery and arthroscopy. The search syntax is outlined in Table 1. Search database used were: MEDLINE^®^, Embase^TM^, CINAHL^®^ (Cumulative Index to Nursing and Allied Health Literature) and the Cochrane Central Register of Controlled Trials (CENTRAL). Search date intervals included were from their year of inception to the last week of September ending 28 September, 2014 and limited to English language and humans. PubMed was used as the primary source and search engine for MEDLINE and MeSH (Medical Subject Headings) were used. The search terms included were: surgical trainees, surgery, orthopaedics, computer simulation, arthroscopy, skills and assessment. Alternative keywords, term variations and search strategy are outlined in Table 1. The same terms were used as search keywords for the other literature sources.

**Table 1.** Database, search terms and search strategy.

Database	Search terms	Alternative terms and search strategy
PubMed EMBASE CINHAL Cochrane- CENTRAL	Surgical Trainees Surgery Orthopaedics Computer simulation Arthroscopy Skills Assessment	Specialty trainees OR Specialist registrar OR Junior Doctors OR Residents AND Surgery OR Surgical AND Orthopaedics OR Orthopaedics AND Simulator OR Computer Simulation OR Computer Interface AND Arthroscopy OR Arthroscopic AND Skills OR psychomotor skills OR performance AND Assessment OR Evaluation

The primary inclusion criteria for this review were studies, which looked at the use of computer simulators in arthroscopic surgical education among surgeons in training. Studies which looked at the use of haptic technology, and computer generated outcome data were included. This review focused on educational outcomes of training as surrogate for objective measurement of performance. Virtual reality motion analysis has demonstrated validity and reliability as an assessment tool for surgical skills across various specialities including arthroscopy [15, 23–25]. Objective procedure-based assessment is widely adopted for the assessment of intraoperative arthroscopic techniques; this has been validated and adapted by the Joint Committee on Surgical Training and Orthopaedic Speciality Advisory Committee [26–29]. The criteria for eligibility were therefore derived from the research parameters outlined below:

Participants – limited experience orthopaedic surgical trainees, speciality trainees or surgical residents.
Intervention – computer simulated arthroscopic surgery of the knee, including both diagnostic arthroscopic manoeuvres and simple therapeutic intervention such as resection of a torn meniscus.
Comparison – standard case-based training under supervision of more experienced surgeons.
Outcomes – transfer validity, 3D motion analysis and performance measured by standard assessment tools.

As delineated by the research question, the focus of this review was on transfer of arthroscopic skills to the operating theatre and retention of these skills overtime. The principal exclusion criteria from this review were: case reports, technical reports, review articles and abstract only or conference proceedings publications. Equally, studies which looked at cadaveric models only, biomechanics, protocol and study design, and computer-assisted diagnosis or surgery were excluded. Similarly, studies on simulator-based assessment, validation of assessment tool, and studies without a comparison group, were excluded.

**Figure 1.**
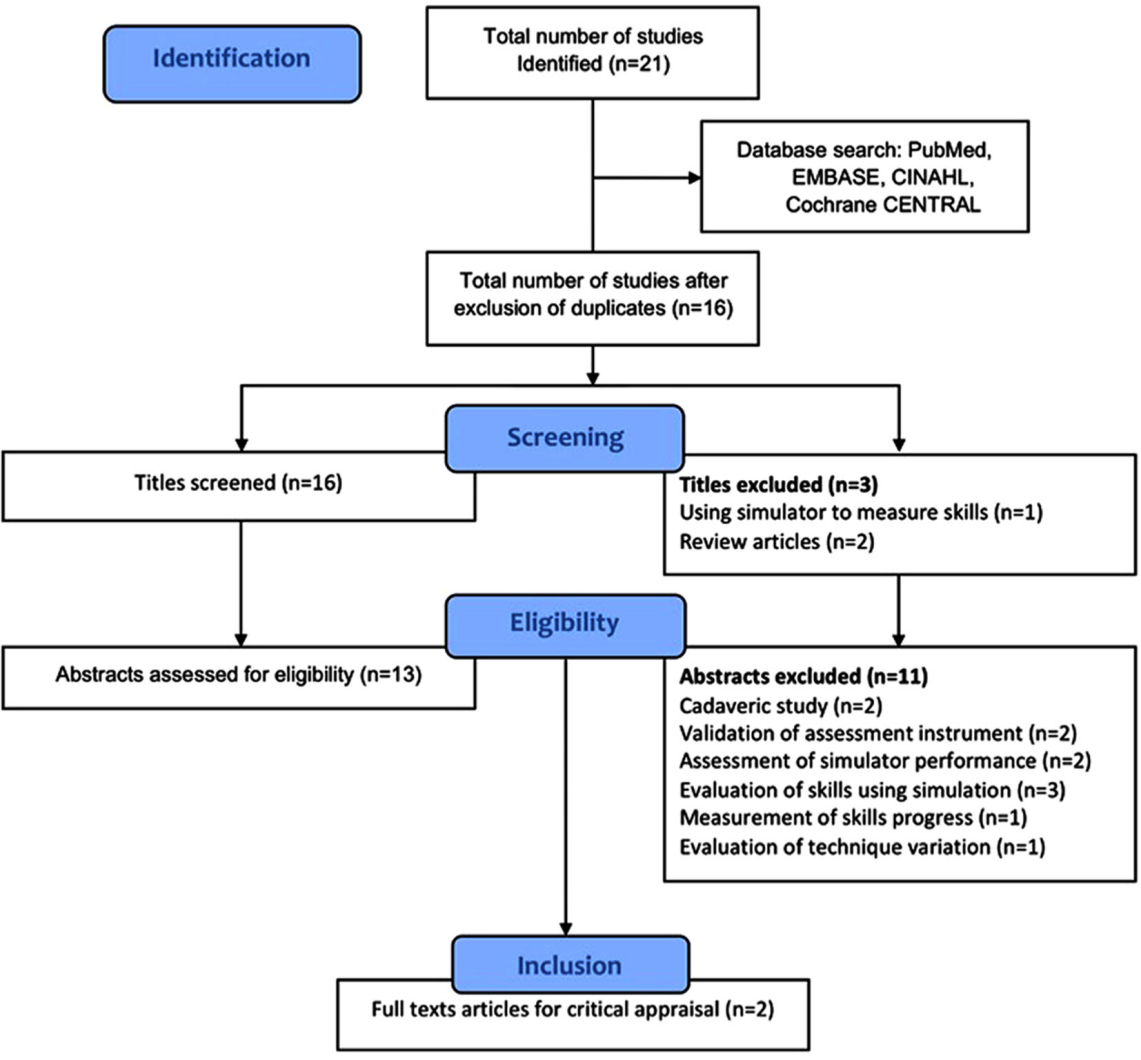
Flow diagram of the systematic literature search.

A rigorous systematic search was performed using the criteria outlined above. References were transferred into Endnote referencing software and duplicates were discarded. Firstly titles and abstracts were reviewed for relevance according to the research question. The remaining studies were analysed in their entirety. Access to full-text articles was obtained from Athens (Eduserv^©^) and Warwick University library. References of full texts were also reviewed to identify any other potentially relevant study. The acquisition process of articles is outlined as a flow diagram in Figure 1. The final studies were reviewed according to study design, analysis and interpretation as well as validity of results. Eligible studies were critically appraised with narrative data synthesis relevant to the study design to identify the best evidence. The latter followed a systematic approach considering participant, intervention, comparators and outcomes as an initial assessment of research question. Criteria derived from the Consolidated Standards of Reporting Trials (CONSORT) were also used for screening study design and conduct [30]. A critical appraisal checklist was drawn from the validated Critical Appraisal Skills Programme (CASP) and used alongside the final two articles included in the review [31, 32].

## Results

A total of 21 studies were generated from all the database searches. This yielded 16 titles for initial screening after removal of five duplicates. Following initial screening and application of the inclusion criteria three titles and 11 abstracts were excluded from the review. The numbers and reasons for exclusion following rigorous tiles screening, abstracts’ assessment and review of full texts are outlined in the flow diagram (Figure 1). The results of this systematic search therefore generated a total of two studies addressing the key questions and suitable for appraisal. These studies consisted of randomised controlled trials, level of evidence I [33]. The final studies are further summarised in Table 2.

**Table 2.** Summary of the studies reviewed.

Study	Participants	Intervention	Comparison	Outcome measures	Principal results
Howells et al. [34]	20 orthopaedic trainees; Less than 2 years experience	Group 1, *n* = 10; Computer simulator training	Group 2, *n* = 10; No additional training	Motion analysis; OCAP*/PBA† assessment; Technical global rating scale 1–5	Simulator training improved performance in the operating theatre
Jackson et al. [35]	19 orthopaedic residents at least 20 diagnostic arthroscopies.	Repeat task 1/months for 5 months; Repeat task once only	Did not repeat the task	Motion analysis on simulator; Assessment of repair by experienced surgeon	Skills retained at 6 months; Improved performance with regular simulator training.

* Orthopaedic Competence Assessment project

† Procedure-based assessment.

Howells and colleagues [34] studied the transfer of arthroscopic skills from the simulator to the operating theatre. Twenty orthopaedic trainees considered novices in diagnostic arthroscopy of the knee were randomised either to simulator training or to no additional training. Both groups received a standardised assessment in the operating theatre. This study showed that the group trained on simulators achieved better scores on the Orthopaedic Competence Assessment Project and Objective Structured Assessment of skills. They also showed a significant improvement in task-specific 3D motion analysis.

In this study closed randomisation methods with clearly established eligibility criteria were reported. The trial addressed a clearly focused issue, and assessed trainees belonging to the same institution hence minimising selection bias. In addition, the study was completed and no loss to follow-up was reported. The primary outcome measure was defined; however, there was no power calculation, a small sample size and unclear definition of the extent of prior arthroscopic experience. Furthermore, the outcome measures used were poorly validated. The assessment of competence by a senior surgeon may have potentially introduced observer’s bias and the assessment method had not been previously validated for the assessment of arthroscopic skills. The results lacked report of confidence intervals; and the untrained group had a narrower interquartile range in both results. This study in effect demonstrated improved acquisition of skills when comparing simulator training to no additional training.

Jackson and colleagues [35] investigated the retention of arthroscopic skills following simulation training and assessment in the operating theatre. Nineteen orthopaedic trainees with an experience of at least 20 diagnostic knee arthroscopies were selected. At baseline all the trainees received a standard simulator-based arthroscopic meniscal repair training. Participants were randomly assigned into Group A: performed the same task once per month during the study period, Group B: performed the task once only at three months, and Group C: did not perform the task again. Groups A and B showed improvement in task performance measured by motion analysis. Group C showed no improvement but no deterioration in 3D motion analysis parameters after a six-month break.

This study utilised well-defined eligibility criteria and a distinct training protocol. In addition, it addressed a focused issue with a clear definition of all intervention and comparator groups. The participants received a standardised objective 3D motion analysis. The study sample was a good representation of trainee population and level of experience. Furthermore, the trial was completed with no report of loss to follow-up and all the clinically important outcomes were considered. Nevertheless, the authors did not report whether assessors were blinded to training status. Several other limitations can be highlighted from this article. A power calculation was not reported to establish the required sample size and clinically relevant differences in the outcomes. The primary outcome measure was not clearly defined and no report on whether any of the outcome measures were validated. The authors determined prior experience based on previous reports by candidates but no objective assessment of prior experience was identified as part of the study protocol. Similarly, the authors did not address the potential bias from the influence of other surgical experience gained during the study period.

## Discussion

The annual report of 2009 from the Chief Medical Officer highlighted the importance of simulation in clinical training [36, 37]. The interests in simulation came with the promise of a safer healthcare delivery and better acquisition of technical skills and human factors training. As a result the facilities required to provide this training have developed widely across the United Kingdom. This expansion is reflected in the research seeking to validate the various simulation-training modalities. With the demand of high-level technical skills and strict safety protocols, surgical specialities stand to potentially benefit greatly from this development. The use of computer simulation in arthroscopy has until recently remained the remit of laboratory-based training courses [23, 38, 39]. The previous body of research demonstrated content validity and reliability of computer-based simulation of arthroscopic training; however, this evidence remained anecdotal with no significant evidence of link with real life situation within the operating theatre [38, 40]. It also appears that such development is likely to find a more favourable environment, given the current emphasis of objective standardised methods of training and assessment introduced in orthopaedic education [27, 29]. This review aimed to evaluate the evidence for improving arthroscopic surgical skills using simulation training among orthopaedic trainees and whether these skills are transferrable to the operating theatre and retained over time. A systematic search and appraisal of the literature revealed two articles which sought to establish that surgeons trained on computer simulator for knee arthroscopy demonstrate better transfer and retention of psychomotor skills when evaluated with standard competence-based assessment and objective measures of motor skills.

The studies presented in this review sought to validate the use of computer simulation in arthroscopic training. Both studies were randomised single blind trials and used similar types of simulators and outcome measures. There were however discrepancies in their objectives and results. The study by Howells et al. demonstrated improved performance of basic knee arthroscopic tasks following a fixed period of simulator training. At the time of its publication this study was the only research looking at transfer and predictive validity of simulator arthroscopic training. The study by Jackson et al. also sought to demonstrate transfer and predictive validity of simulator training. This study had the particularity of looking at the retention of psychomotor skills over a pre-defined period of time. The authors not only demonstrated an improved performance of knee arthroscopic tasks but also no deterioration in the levels of skills attained following a period of six months. In addition, the two studies succeeded in highlighting the importance of 3D motion analysis using computer simulators in the assessment of technical skills. Components of evaluation such as time to completion of task, distance travelled by instruments and incidence of instruments collisions were associated with the highest validity and reliability of overall arthroscopic skills assessment. The two articles contribute to the body of evidence on internal validity and consistency of arthroscopic computer simulators shown in previous studies [18, 39].

Howells et al. used an objective assessment of technical skills tool, a previously validated scale which was shown to correlate with motion analysis [41]. This was aimed to counteract the fact that only the simulator group was assessed using the objective computer-based 3D motion analysis. This study showed that one of the trainees had a poor correlation between simulator and theatre performance. The authors as a result recognised the heterogeneity, which trainees can exhibit both in terms of capability and speed of learning psychomotor skills. Howells et al. were therefore able to show acquisition of basic knee arthroscopic skills in a group of novices. In contrast, Jackson et al. were the first authors to demonstrate the transfer and long-term retention of arthroscopic skills among orthopaedic trainees. The authors clearly demonstrated the presence of a learning curve in the development of arthroscopic meniscal repair skills using computer simulators. Previous similar research has focused primarily on laparoscopic skills and surgical skills training among medical students [13, 14, 42]. Equally, this study reinforced the knowledge that a higher volume of cases was associated with better performance. Nevertheless, the differing levels of task performance achieved by trainees detract from the belief that a minimum number of task repetitions is required to achieve competence [43].

The two articles discussed here present multiple and recurrent limitations in their design, conduct and interpretation. Both studies had small sample sizes, which mitigate the generalisability of their results. The two groups of authors concentrated on knee arthroscopic skills, which render the results difficult to extend into other joint conditions commonly treated with arthroscopic procedures. Even though, previous studies showed internal validity of computer simulator in shoulder arthroscopic training [39]. In addition, both studies presented challenges in objectively delineating the extent of pre-study arthroscopic skills among participants resulting in selection bias. This type of bias can also propagate during the study period, unless strictly controlled; trainees can continue to develop technical skills through other sources. Howells et al. utilised outcome measures not previously validated for simulation arthroscopic training and relied on a single assessor in the operating theatre. Although these authors demonstrated improved skills in the operating theatre, the results were extracted from a comparison of additional training versus no training. The results presented by Jackson et al. were mitigated by the lack of a valid objective assessment tool utilised in the operating theatre. These authors recognised the limitations in part of their results due to inability to differentiate between incomplete task due to device failure or to task failure by the participant.

It has become clear that simulation plays an important role in the creation of the next generation of orthopaedic surgeons. The current climate of working time directives and expectations from the general public support the case for simulation training. The traditional model of “see one, do one, teach one” has therefore become no longer tenable, and it is considered inconsistent with the good clinical practice guidelines established by the General Medical Council [44]. In addition, modern-day simulators can open new spheres to trainees with exposure to variations in normal anatomy and less frequent clinical conditions. The repetitive pattern of simulation training can even attract advocates of the traditional apprenticeship model, which created the previous generations of surgeons who learned through a successive undertaking of the same task under close scrutiny of their senior colleagues. This review highlights the possibility of marrying simulation with clinical reality as a way forward from the conventional computer simulator. Further developments are still needed however to help refine the use of simulation to meet the exact needs of individual trainees and trainers. The work by Howells et al. and Jackson et al. opens the scope for further research into the practical implications of arthroscopic simulation training. Despite their important contribution these authors have not addressed other pertinent questions. Does arthroscopic simulation help the acquisition of professional skills such as teamwork and operating theatre etiquette? What are the cost implications for the use of simulation in the development of psychomotor arthroscopic skills? These questions require the attention of the orthopaedic education community and should form part of any future research direction. There is limited research available on the cost-effectiveness of computer-based simulation training and only restricted to other surgical specialities [45]. Current evidence is based on small studies and limited to abdominal surgical specialities [46]. Although the cost implications will remain a valid argument, the overall savings in relation to improved operative outcomes and patients’ satisfaction can offset the financial impact of added training [47]. The implication of arthroscopic simulation on curriculum design and the perception of trainers have not been investigated to date. Studies of this nature remain limited and only focused on other surgical specialities. Coulter and Brennan investigated the experiences and perceptions of surgical simulation training among neurosurgeons [48]. These authors reported that 80% of neurosurgeons considered simulation a valuable addition to training. They also found that the majority of experienced surgeons used simulation outside the clinical settings and had raised concerns over transferability of skills. Similar issues were raised about training in conditions considered of higher complexity. In contrast, patients demonstrate a greater acceptance of simulation training according to a previous survey [49]. These authors concluded that the conventional situation in which patients took a passive role in medical education is rapidly disappearing [49]. It is also important here to consider the opinion of trainees on arthroscopic simulation. Nevertheless, research of this type remains unavailable. Cleave-Hogg and Morgan analysed students’ perceptions on anaesthesia simulation training [50]. These authors reported 88% positive learning experience among participants. In this study 83% of participants felt that simulation provided them with a realistic clinic experience [50].

Further studies are therefore needed to investigate other training units, and other joints. Such research will also need to address other simulation-training models such as cadaveric labs as a potential stepping-stone for the transfer of skills from the computer simulator to the operating theatre. Future studies will need to employ validated outcomes measures pertinent to transfer and retention of skills, as well as longer-term evaluation of surgeons trained on simulators. Such research will also need to adjust for skill levels and clearly define the nature of on-going additional training the participants receive during the study period. In addition, the introduction of validated pre- and post-training evaluation tools can help reduce the risk of selection bias. Equally, a more robust link between the operating theatre and the simulation lab needs to be introduced in future studies, this can be performed with objective evaluation of trainees within real clinical setting prior to and following simulation training. A clear distinction between studies investigating the utilisation of computer simulators as an assessment tool or a training tool needs to be established. With the advent of personal communication devices the introduction of video assessment of trainee performing a surgical procedure can also help optimise the current model of procedure-based evaluation of surgical trainees. For instance, early work within other surgical specialities using Google Glasses™ as a training instrument promises to deliver satisfactory outcomes [51]. Ultimately, a careful selection and appraisal of available simulation technology should form an integral part of all future researches. The introduction of such standardised high fidelity computer simulators should help deliver more pragmatic studies with results that are easier to interpret.

## Conclusions

Haptic technology and computer generated outcome measures have been shown to be valid training and assessment tools. This review helped highlight the contribution of the two studies in terms of internal validity and consistency of arthroscopic skills training using computer simulators. The two articles appraised add evidence of transfer validity to arthroscopic simulator training, and help build the case for further research in the field. Future studies of higher quality are therefore needed in order to address the limitations raised in this review. Due to the complex nature of skills and the impact on health service and patient safety, stricter research criteria need to be applied. Future work needs to incorporate assessment of cost-effectiveness, perceptions from trainers and trainees as well as the impact such training can have on curriculum design. The technology will inadvertently continue to progress alongside the availability of increasingly realistic simulators. It is therefore likely that continued research will further validate the field of simulation making it an integral part of clinical training and assessment. Equally, simulation will need to remain under strict professional scrutiny, to which all other aspects of clinical training are subject. Consequently, establishing a firm link between simulation and real clinical situations will be pivotal in sustaining this development. Future research, taking into consideration the vital elements raised from appraising the best available literature, will help provide better answers on the transfer validity of arthroscopic simulation training.

## Conflict of interest

The authors declare no competing interests. No external funding sources were received.
